# Predictive *vs.* Empiric Assessment of Schistosomiasis: Implications for Treatment Projections in Ghana

**DOI:** 10.1371/journal.pntd.0002051

**Published:** 2013-03-07

**Authors:** Achille Kabore, Nana-Kwadwo Biritwum, Philip W. Downs, Ricardo J. Soares Magalhaes, Yaobi Zhang, Eric A. Ottesen

**Affiliations:** 1 RTI International, Washington, District of Columbia, United States of America; 2 Neglected Tropical Diseases Control Programme, Ghana Health Service, Accra, Ghana; 3 University of Queensland, Infectious Disease Epidemiology Unit, School of Population Health, Brisbane, Australia; 4 Helen Keller International, Regional Office for Africa, Dakar, Senegal; London School of Hygiene & Tropical Medicine, United Kingdom

## Abstract

**Background:**

Mapping the distribution of schistosomiasis is essential to determine where control programs should operate, but because it is impractical to assess infection prevalence in every potentially endemic community, model-based geostatistics (MBG) is increasingly being used to predict prevalence and determine intervention strategies.

**Methodology/Principal Findings:**

To assess the accuracy of MBG predictions for *Schistosoma haematobium* infection in Ghana, school surveys were evaluated at 79 sites to yield empiric prevalence values that could be compared with values derived from recently published MBG predictions. Based on these findings schools were categorized according to WHO guidelines so that practical implications of any differences could be determined. Using the mean predicted values alone, 21 of the 25 empirically determined ‘high-risk’ schools requiring yearly praziquantel would have been *undertreated* and almost 20% of the remaining schools would have been treated despite empirically-determined absence of infection – translating into 28% of the children in the 79 schools being undertreated and 12% receiving treatment in the absence of any demonstrated need.

**Conclusions/Significance:**

Using the current predictive map for Ghana as a spatial decision support tool by aggregating prevalence estimates to the district level was clearly not adequate for guiding the national program, but the alternative of assessing each school in potentially endemic areas of Ghana or elsewhere is not at all feasible; modelling must be a tool complementary to empiric assessments. Thus for practical usefulness, predictive risk mapping should not be thought of as a one-time exercise but must, as in the current study, be an iterative process that incorporates empiric testing and model refining to create updated versions that meet the needs of disease control operational managers.

## Author Summary

The challenge of accurately mapping schistosomiasis is a daunting one – particularly because of the highly focal distribution of the disease. Ideally, of course, each specific treatment area would be assessed for infection prevalence and then treated appropriately based on guidelines of the World Health Organization. In practice, however, this is not possible, and a variety of short-cutting techniques have been developed to meet these mapping needs, including geospatial predictive mapping. This paper assesses the accuracy of model-based geostatistics (MBG) predictions for determining treatments projections in Ghana by comparing previously published data using MBG predictions with empirically derived prevalence values for schistosomiasis from school surveys completed at 79 sites. We found that using predictive mapping alone would not have provided reliable information for mass drug administration (MDA) planning – resulting in overtreatment in some areas and most importantly under-treatment in areas that needed it most. Based on our findings, predictive risk mapping cannot be a one-time exercise but must instead be a process that incorporates empiric testing and model refining to create optimised spatial decision support tools that meet the needs of disease control operational managers.

## Introduction

Schistosomiasis inflicts a high socioeconomic burden in the tropics and subtropics, especially in sub-Saharan Africa [1]. The disease is caused by infection with trematode *Schistosoma spp*., transmitted through fresh water snails whose distribution is limited and particularly sensitive to environmental changes, including those caused by altering water resources. Transmission of schistosomiasis is focal, and the spatial heterogeneity across specific geographical areas reflects various human, snail host, and ecological factors [2].

Until 2008, the prevalence of schistosomiasis in Ghana had not been fully documented [3], though it was recognized that *Schistosoma haematobium* (urinary schistosomiasis) is more prevalent than *S. mansoni* in the country [4]. Potentially endemic communities lacked a clear designation of eligibility for mass drug administration (MDA) with praziquantel (PZQ) – not only who among the total population of school-age children (SAC) and high risk adults (HRA) should be treated, but also how often such treatment should be given following the preventive chemotherapy (PCT) guidelines recommended by the World Health Organization (Table 1), [5], [6]. Prior to 2007 most schistosomiasis control in Ghana focused on snail vector control and was mainly undertaken in areas within the Volta River basin, but in some areas non-governmental organizations (NGOs) distributing de-worming drugs to treat soil-transmitted helminth (STH) infections also distributed PZQ in those communities endemic for both schistosomiasis and STH.

**Table 1:** WHO recommended treatment strategy for schistosomiasis in preventative chemotherapy.

Prevalence* in school-age children (SAC)	Treatment Group	Target Group	Treatment Freq.
≥50%	A (High)	Treat all SAC and adults at risk	Once every year
≥10% and <50%	B (Medium)	Treat all SAC and adults at risk	Once every 2 years
≥1% and <10%	C (Low)	treat all school-aged children	Once every 3 years (or twice during primary schooling age)

* *determined by parasitological methods [Source* [*5*], [*6*] *]*.

Schistosomiasis prevalence mapping is essential to identify and enumerate target populations for MDA by establishing prevalence of infection among SAC in a defined implementation unit (IU) [6]. For *S. haematobium,* prevalence can be determined by sampling and assessing the presence of infection among SAC in schools using urine filtration, dipstix, or a standard questionnaire for history of haematuria; for *S. mansoni* and STH, stool examination using the Kato-Katz method is most commonly used [6], [7]. Results of these prevalence surveys are related to the international guidelines (Table 1) to determine the specific treatment regimen and target population within the IU.

Because, however, it is impractical to assess the prevalence of infection in every community of an endemic area [8], previous work has employed disease risk mapping to predict prevalence of parasitic infections at unsampled locations, including the use of model-based geostatistics (MBG) to assess the risk of *S. haematobium* within district level IUs [9]–[11]. In Ghana, MBG was applied to parasitologic data collected in nationwide school-based surveys conducted during 2008, to predict prevalence [11], [12] and intensity [13] of infection throughout the country. The findings confirmed the earlier assessment that the geographical distribution of *S. haematobium* in Ghana was highly heterogeneous and more widespread than that of *S. mansoni* [11].

With the aim of evaluating the predictions of the 2008 predictive prevalence maps, we took advantage of additional, available school-based data collected during subsequent surveys conducted in 2010 in both previously sampled and un-sampled districts. This study compares for *S. haematobium* infections, Ghana's most widespread schistosome infection, predictions from the spatial modelling with the empiric results gathered during the 2010 survey. Further, we assessed the practical implications of using either the predictive model or the empiric observations to determine the appropriate treatment regimen (and, thus, the appropriate utilization of the PZQ needed to treat the children in these schools) during a six-year projected period, the newly recommended duration of treatment programs before determining the impact of the intervention and reassessing subsequent programmatic strategy [5].

## Methods

### Ethical statement

Ethical approval for the survey in 2008 was obtained from Imperial College Research Ethics Committee UK [8] and the Ghana Health Service Ethical Review Committee in Ghana as described previously and for the survey in 2010 it was obtained from the Ghana Health Service Ethical Review Committee. Before the survey, the official letters were sent by the Ghana Health Service to the Regional and District health and educational authorities to inform the activities and to request support. On the day of survey, the survey team explained the activities to and obtained verbal consent from the village head, the school headmaster, the parent-teachers association and the local health authorities. Verbal consent was also obtained from parents/guardians of all children involved in the study, and those who did not want their children to participate informed the school authorities. Children who participated were given an explanation of the data collection activities and were free not to participate if they so chose. Written consent was not obtained and verbal consent was approved by the ethics committees involved because the surveys were considered part of routine disease mapping activities of the Ghanaian national NTD control program and performed by the Ghana Health Services of the Ministry of Health.

### Focus on *S. haematobium*

In *Ghana S.mansoni* is concentrated in only a few focal areas, which were not included in any of the 79 sites selected for this study. For this reason only *S. haematobium* prevalence was considered in the present analyses.

### Predictive prevalence map of *S. haematobium* infection for 2008

The 2008 *S. haematobium* predictive risk map developed for Ghana was based on a nationwide parasitological survey carried out in 77 schools during March to May 2008. As described in detail elsewhere [8], schools were purposively selected through a spatial stratification procedure where rural schools and schools adjacent to Volta Lake were twice as likely to be sampled. The number of schools selected from the districts was calculated to be proportional to the area of the districts; sixty children from each of the selected schools were recruited into the survey and examined for infection with *S. haematobium*. This data was incorporated in a binomial MBG model together with environmental data known to be associated with disease distribution, *e.g.* NDVI (Normalized Difference Vegetation Index, a proxy for rainfall), land surface temperature and distance to water bodies, and a geostatistical random effect to produce a spatial predictive risk map for *S. haematobium* based on studies of children aged 9–15 years in all districts of Ghana [8]. Predictions were made at the nodes of a 0.1×0.1 decimal degree grid (approximately 12 km^2^).

### Additional school-based parasitological survey

Between April and July 2010, 123 additional schools that were not sampled during 2008 were surveyed by the Ghana Health Service in 92 districts with support from the U.S. Agency for International Development's NTD Control Program. Selection of survey sites was based on criteria from previous survey work conducted in 2008 [8] and on proximity to susceptible sources of water. Fifty children between the ages of 9–15 years (classes 4–6) were randomly selected from within each selected school and examined for *S. haematobium.* The observed prevalence of *S. haematobium* infection was used to categorize the endemicity in schools according to the 2006 WHO guidelines [6]. The protocol used for parasitological examination required that each child included in the survey provide a single urine sample using a plastic container. Urine samples were examined using the standard filtration method; urine samples were collected in 60 ml plastic screw-cap vials, between 10.00 and 14.00 hours. Each urine sample was agitated to ensure adequate dispersal of eggs, 10 ml of urine was filtered through a Nuclepore filter and the number of *S. haematobium* eggs counted under a light microscope by experienced examiners. Individual infection with *S. haematobium* was recorded as number of eggs per 10 ml of urine.

### Comparative study

Since the completion of the 2008 survey, 79 schools among the 123 schools surveyed in 2010 were never targeted for treatment with PZQ. As schistosomiasis prevalence in relatively stable environments remains remarkably constant in the absence of treatment and improvement of sanitary conditions [5], data from these 79 schools, which represent 61 districts out of a total of 170 districts, were used to compare predicted and empiric prevalence assessments (Table 2). The 79 schools were categorized in two ways. Most simply, according to the WHO recommended prevalence thresholds [6] based on the individual school results. More formally, the locations of the 79 schools were linked to the predictive prevalence maps from Soares Magalhaes et al. (2011) in a geographical information system.

**Table 2:** Schistosoma haematobium infections in Ghana 2010.

2010 Surveys	Schools (Districts)	Tested 9–15 yrs.	S.haematobium infection (%)
Number of sites surveyed	123 (92)	6,250	33.7%
Number of sites not treated	79 (61)	3,950	31.9%

To determine the discriminatory performance of the model predictions relative to observed prevalence the area under the curve (AUC) of the receiver operating characteristic was used [14]. An AUC value of >0.7 was taken to indicate acceptable predictive performance. The full predictive distribution at each pixel was accounted for, and then the mean predictive prevalence, the median predictive prevalence, the 97.5% Credible interval (CrI) predictive prevalence, and the 2.5% CrI predictive prevalence were estimated. The observed *S. haematobium* prevalence was categorized into three WHO prevalence thresholds and comparisons were made with the mean, median, 97.5% Crl, and 2.5% Crl. An AUC lower than 0.7 indicated a poor discriminative capacity in distinguishing between observed and predicted prevalence, whereas an AUC from 0.7–0.9 indicated a reasonable capacity and >0.9 indicate a very good capacity [14].

The implications of using the empiric *vs.* modelling strategies to determine the WHO-guideline *S. haematobium* treatment categories were examined using the total numbers of school children in the 79 surveyed schools. The annual number of SAC to be targeted for treatment in each school was determined using the 2008 district mean predictive prevalence map and the 2010 observed prevalence categories, over a 6-year period of projected program implementation. The differences between the two methods to identify the number of SAC to be treated provided a measure by which to compare the projected number of SAC targeted for treatment over a 6-year period of program implementation and to identify how many children would be under-treated or over-treated depending on whether the predicted or empiric prevalence levels were utilized with the WHO guidelines.

## Results

### Determination of prevalence categories

**Figure 1:**
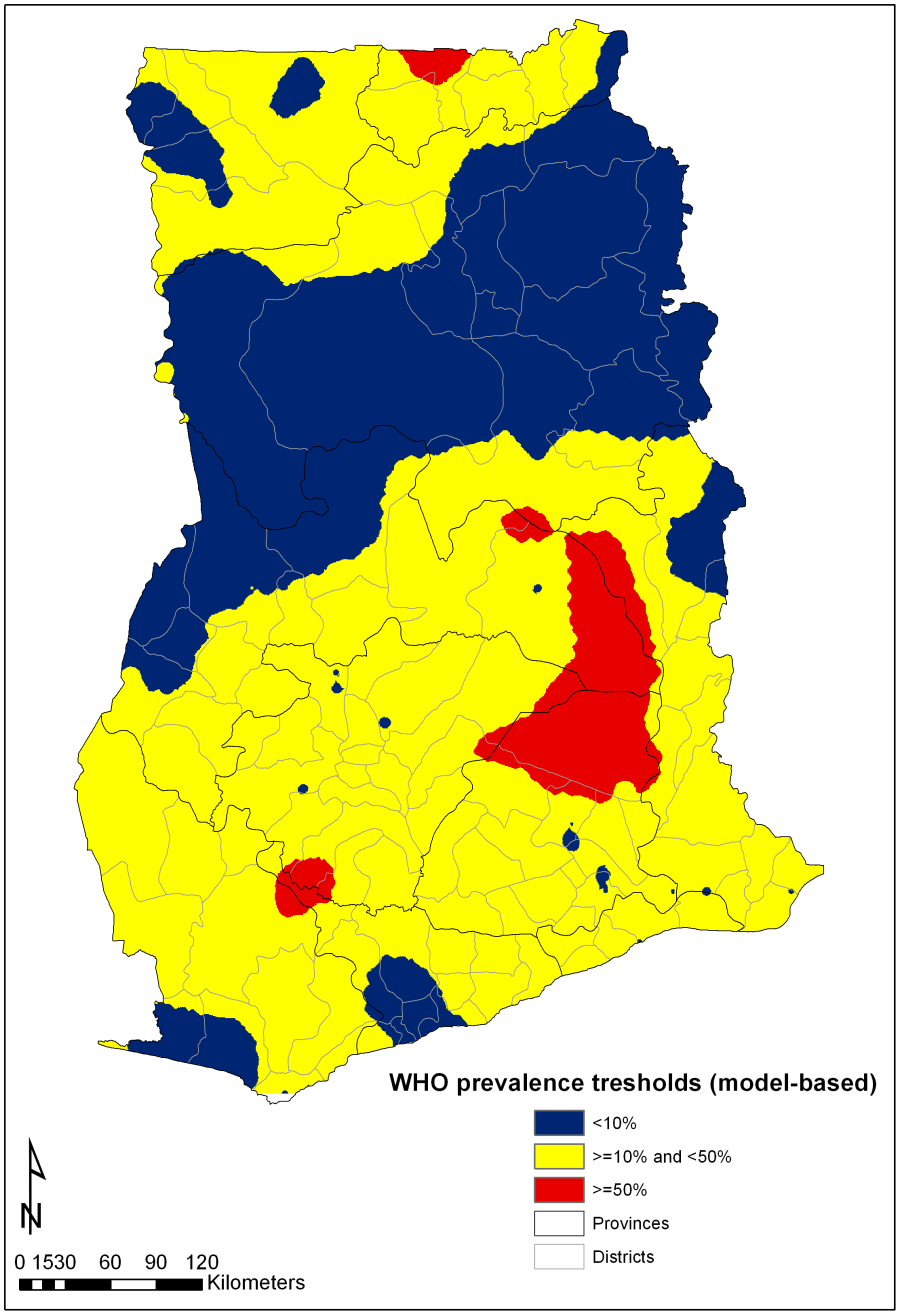
Model-based stratification of schistosomiasis prevalence, 2008.

Figure 1 summarizes the Model-based stratification of schistosomiasis prevalence according to the continuous spatial predictive risk map for *S. haematobium* in 2008. The AUC analysis revealed that the predictive map had acceptable predictive capacity to distinguish areas of high (≥50%) prevalence of *S. haematobium* in 2008, in particular when the mean predictive value for the district was used (Table 3). However, these values show very poor capacity to distinguish between areas of medium (≥10% and <50%) and low (<10%) observed prevalence of infection for 2008. Based on these results the mean predictive value for districts were used as a comparison for observed single-location data.

**Table 3:** Observed prevalence threshold vs. predicted prevalence using area under the curve (AUC) analysis*.

Predicted prevalence	Observed Prevalence > = 50% (High)	Observed Prevalence > = 10% and <50% (Medium)	Observed Prevalence > = 1% and <10% (Low)
Mean predicted	0.70 (0.58,0.82)	0.55 (0.42,0.68)	0.27 (0.14,0.39)
50% Cr predicted	0.70 (0.57,0.82)	0.55 (0.42,0.68)	0.27 (0.15,0.40)
97.5% Cr Predicted	0.68 (0.56,0.81)	0.56 (0.42,0.69)	0.27 (0.15,0.40)
2.5% Cr Predicted	0.61 (0.47,0.74)	0.52 (0.401,0.64)	0.38 (0.27,0.50)

* *An AUC lower than 0.7 indicates a poor discriminative capacity in distinguishing between observed and predicted prevalence, whereas an AUC from 0.7–0.9 indicates a reasonable capacity and >0.9 indicates very good capacity*.

The prevalence categories determined by the 2008 predictive prevalence maps for *S. haematobium* indicated that of the 79 selected schools for comparison 17 (22%) where located in districts in which the mean predicted prevalence for the district was estimated to be <10% (low endemicity), 58 (73%) in districts with predicted prevalence between 10% and 50% (moderate endemicity), and 4 (5%) in districts with predicted prevalence of ≥50% (high endemicity).

**Figure 2:**
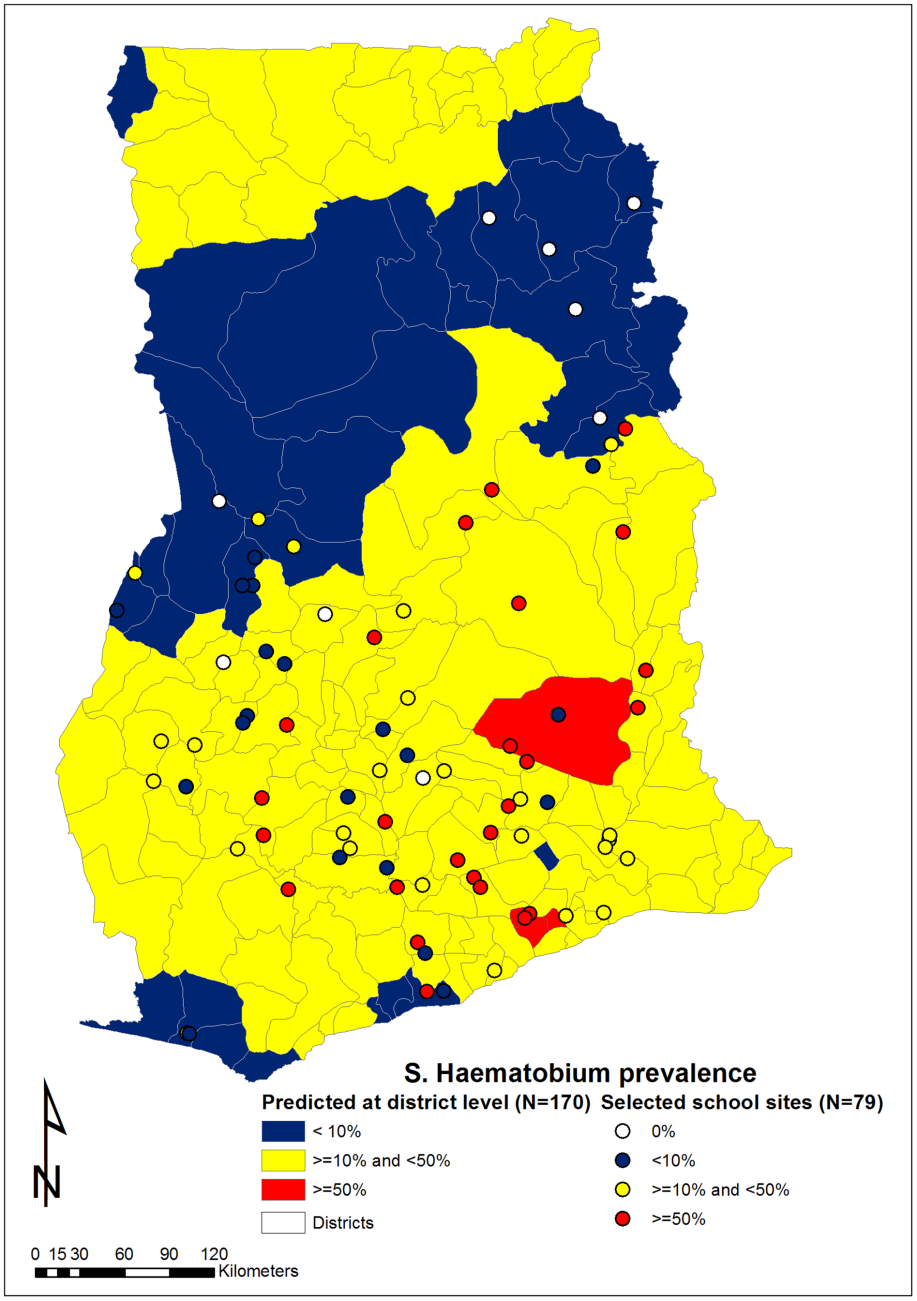
Distribution of schools selected in the 2010 schistosomiasis survey.

Based on the results of the 2010 school-based surveys, these same 79 schools were categorized according to their observed prevalence: 9 (11%) with 0% prevalence of *S. haematobium* infection (non-endemic); 20 (25%) with 1–<10% prevalence (low endemicity); 25 (32%) with 10–<50% prevalence (moderate endemicity); and 25 (32%) having ≥50% prevalence (high endemicity). Figure 2 summarizes the geographic distribution of selected schools and the mean aggregated predicted prevalence for districts according to the spatial predictive risk map for *S. haematobium* in 2008. The difference between the prevalence categorizations according to the observed prevalence and the predicted prevalence map is shown in Table 4. Compared to the empiric findings, the mean predictive mapping resulted in appreciable overestimations of *S. haematobium* endemicity for some of these 79 schools and appreciable underestimations for others.

**Table 4:** Levels of *S. haematobium* prevalence in schools as determined by either predictive mapping or empiric observations (N = 79).

Predicted prevalence in Schools (mean)	No. Schools with observed prevalence (0%)	No. Schools with observed prevalence (>0%–<10%)	No. Schools with observed prevalence (≥10–<50%)	No. Schools with observed prevalence (≥50%)
0%	0	0	0	0
>0%–<10%	6	3	4	1
≥10–<50%	3	16	21	21
≥50%	0	1	0	3
Total	9	20	25	25

### Practical implications of different determinations of prevalence

To understand the implications of the differences in prevalence shown in Table 4, the WHO recommended categories and treatment guidelines (Table 1) were applied to these 79 schools – a total of 18,148 students (Table 5). It can be seen that both Category A (high prevalence) and the ‘zero-prevalence’ schools were *underestimated* by the predictive mapping, while Categories B (medium) and C (low) were *over-estimated* compared to the empiric assessments. During the 1^st^ year 16,015 SAC were targeted for treatment based on the results of the empiric school-based surveys; all would also have been targeted for treatment as well based on predictive mapping. The predictive model, however, targeted all 18,148 students from the 79 schools for treatment in the 1^st^ year, with the 2,133 ‘extra’ SAC treatments resulting from the children in 9 schools subsequently determined empirically to have zero prevalence of *S. haematobium* in the 2010 school-based survey. These students would not have been slated for treatment based on empiric studies and would be considered as ‘over-treatments’ each year they would be treated. During the 2^nd^ year of treatment, 5,930 students in 25 schools would be targeted for treatment according to the empiric 2010 school based surveys compared to only 818 students from 4 schools based on the predictive mapping results. During the 3^rd^ year of treatment, SAC in both high and medium prevalence areas would be targeted for PZQ. Based on the 2010 school surveys 12,372 students in 50 schools would be targeted for treatment in the 3^rd^ year, while the predictive model calculated 14,280 SAC from 62 schools. This difference is primarily related to the 2,133 SAC not targeted for treatment from schools with zero prevalence according to the 2010 surveys. The estimated target populations during the 4^th^, 5^th^, and 6^th^ years of treatment would continue to differ as seen in Table 3. During this 6-year cycle a total of 62,192 treatments were projected for the 18,148 SAC according to WHO recommendations using the empirically derived data; though 53,030 treatments were called for using the *predicted* prevalence data, this rather small quantitative difference belies important under- and over-treatments projected for the target population.

**Table 5:** Targeted school-age children (SAC) population among sampled schools determined by predicted or empiric methods.

Prevalence in SAC	Treatment Group	# of Schools	Total SAC	YR 1 Rx	YR 2 Rx	YR 3 Rx	YR 4 Rx	YR 5 Rx	YR 6 Rx	Total Rx
**2010 School Based Surveys**										
≥50	A (High)	25	5,930	5,930	5,930	5,930	5,930	5,930	5,930	35,580
≥10 and <50	B (Medium)	25	6,442	6,442	-	6,442	-	6442	-	19,326
<10	C (Low)	20	3,643	3,643	-	-	3643	-	-	7,286
0	Non-endemic	9	2,133	-	-	-	-	-	-	0
**TOTAL SAC Targeted for Treatment**		**79**	**16,015**	**16,015**	**5,930**	**12,372**	**9,573**	**12,372**	**5,930**	**62,192**
**2008 Predictive Mapping**										
≥50	A (High)	4	818	818	818	818	818	818	818	4,908
≥10 and <50	B (Medium)	58	13,462	13,462	-	13,462	-	13,462	-	40,386
<10	C (Low)	17	3,868	3,868	-	-	3868	-	-	7,736
0	Non-endemic	0	0	-	-	-	-	-	-	**-**
**TOTAL SAC Targeted for Treatment**		**79**	**18,148**	**18,148**	**818**	**14,280**	**4,686**	**14,280**	**818**	**53,030**

## Discussion

The challenge of accurately mapping schistosomiasis is a daunting one – particularly because of the highly focal distribution of the disease. Ideally, each specific treatment area (‘implementation unit’) would be assessed for infection prevalence and then treated appropriately based on the WHO guidelines. In practice, however, this is not possible, and a variety of short-cutting techniques have been developed to meet these mapping needs, including geospatial predictive mapping. Such predictive maps, in principle, are not constrained by administrative ‘implementation units’ (district, sub-district, community, school) but can provide prevalence estimates across a continuous landscape [13]. Since the results of geospatial predictive maps vary depending on the techniques applied and variables included in the model, it is particularly important for program implementation that these mapping outcomes be tested and, if necessary modified with the availability of increasingly detailed data.

The approach taken to testing the predictions of an available, well defined geospatial model for schistosomiasis in Ghana [8] was based on the recognition that treatment regimens in endemic areas are determined by examining the school age children for infection and then basing treatment in that area on the findings in these SAC [4]. We recognize that WHO guidelines do not specifically recommend treating at district level as it was done in Ghana. Instead ecological zonation is recommended which might be less prone to differences in the focal distribution of schistosomiasis. However, districts are more often used to guide treatment decisions because of their operational advantages even despite their potential for imprecision when district-level averages are used in areas where schistosomiasis infection varies considerably from community to community.

Rather than extrapolating the findings from the 79 schools studied to larger areas, we restricted our analysis to just the population of the schools empirically assessed in order to minimize variability that would arise from broader extrapolations. While treatment is planned based on prevalence thresholds for school age children (i.e. 5–15 yrs.) the predictive maps available for analysis were constructed for an age group which is known to be at a higher risk of infection (9–15 yrs.). As expected, our results show that the predicted prevalence maps for the subgroup of the population assessed to be at highest risk of *S. haematobium* infection can be good indicators of areas where prevalence is at its highest. However, our analysis shows poor discriminatory capacity of the predictive models for medium and low prevalence thresholds suggesting that the choice of age group for which predictive maps are developed may constitute an important contributor for the discrepancies between observed and predicted prevalence for medium and low prevalence thresholds. This supports our analysis of treatment figures over a 6 year period, which suggests that predicted prevalence for medium and low prevalence thresholds underestimated the number of observed schools with high prevalence (>50%).

Based on our comparative analysis, we concluded that use of the rigorously described predictive model developed in Ghana earlier [8] and aggregating these prevalence estimates to the district level would not, by itself, have given the program the degree of accuracy it would hope for in establishing its schistosomiasis control program. Certain areas that were predicted to be endemic and require treatment, in fact had no infection found when they were evaluated empirically. As mentioned previously, ecological zonation is recommended and a single school by itself does not represent the potential prevalence within the district. However for the schools that were surveyed in 2010 and showed to have no *S. haematobium* infection, treatment would have been an unnecessary challenge and an unnecessary expense in human and drug (praziquantel) resource for the health system. It was estimated that 12% of the students in schools predicted as eligible for treatment did not, in fact, need it. This discrepancy may result from the inherent limitations of the methodological procedures employed in the generation of the predictive risk map for Ghana. While marginal prediction techniques utilise covariate information from each unsampled location, joint prediction takes into account the values of the predictors from the neighbourhood around an unsampled location. Thus, making district-level estimates from a predictive map which employed marginal prediction is likely to yield imprecise measures of prevalence at the district level.

Of even greater concern, however, were the underestimated prevalence rates predicted in certain areas where infection was identified empirically to be heavy enough to warrant yearly treatment of the SAC. Though during the 6-year period of treatment [5] modelled in our study, 85% of the total number of treatments needed for the population based on the empiric observations were called for by the predictive model, the greatest underestimates were for those who needed treatment the most. Indeed, only 1 in 7 individuals in areas where yearly treatment was required would have received it had the treatment regimen been determined solely by the predicted, rather than the empiric, prevalence rates. Furthermore, the discrepancies identified between the aggregated district-level predicted prevalence estimates and empiric prevalence determinations have been calculated only for the effects on the SAC, but their implications extend further. Since the recommendations for treatment of schistosomiasis in populations where SAC prevalence rates exceed 50% are not limited to children but include the HRA populations as well, the underestimates of prevalence in 21 of the 25 high-risk schools by the predictive model imply significant under-treatment of the needy adults in these populations too.

One need only to extrapolate the numbers of schools and endemic areas in this assessment to the total endemic area of Ghana to appreciate just how many people would be undertreated in many areas and how much praziquantel would be expended inappropriately in other areas to recognize the importance of improving our understanding of the most appropriate way to use predictive risk maps to guide treatment delivery decisions, assessment techniques, and modelling approaches to schistosomiasis (in Ghana and elsewhere). While using the current predictive map for Ghana by aggregating the prevalence estimates at the district–level is demonstrably not adequate, the alternative of assessing each school in potentially endemic areas of Ghana is not at all feasible; modelling must be a tool complementary to empiric assessment [15]. In addition, for practical usefulness, predictive risk mapping cannot be a one-time exercise but must instead be a process that incorporates empiric testing and model refining to create newer versions with increasingly accurate predictions. Additionally, in order to be truly useful for program managers as spatial decision support tools in endemic regions, and not to be restricted to academic centers alone, these models must have a ‘front-end’ that is both user-accessible and user-friendly. Modelling necessarily will have an increasingly important role in schistosomiasis control programs and in schistosomiasis elimination in line with the newest WHO guidelines [5]; it is through building on studies, such as the one we've described, that programs will attain to greater success in correctly recognizing and most efficiently addressing the serious problems of schistosomiasis both in Ghana and worldwide.
